# The preschool classroom linguistic environment: Children’s first-person experiences

**DOI:** 10.1371/journal.pone.0220227

**Published:** 2019-08-07

**Authors:** Leydi Johana Chaparro-Moreno, Laura M. Justice, Jessica A. R. Logan, Kelly M. Purtell, Tzu-Jung Lin

**Affiliations:** 1 Educational Psychology Program, Department of Educational Studies, Ohio State University, Columbus, Ohio, United States of America; 2 Crane Center for Early Childhood Research and Policy, Ohio State University, Columbus, Ohio, United States of America; 3 Quantitative Research, Evaluation and Measurement Program, Department of Educational Studies, Ohio State University, Columbus, Ohio, United States of America; 4 Human Development and Family Science Program, Department of Human Sciences, Ohio State University, Columbus, Ohio, United States of America; Fordham University, UNITED STATES

## Abstract

The linguistic environment of the classroom is influential to young children’s language development. To date, however, literature on the linguistic environment of child-care centers has largely examined teacher practices or children’s aggregate environment, overlooking the child’s first-person experiences and differentiated experiences within the classroom. In this study we used a new method in the educational setting that captures the learner’s perspective: head-mounted cameras. Thirteen children in one preschool classroom wore a head-mounted camera to capture their first-person experiences in one morning session, including interactions with others and the features of the child-directed speech (CDS) addressed to them. Results revealed that, from children’s personal view, the linguistic environment of the classroom is more dynamic from what previous studies have reported. Children interacted for longer with their teachers than their peers and heard more CDS from them, but for some children peers served as an additional source of language. Further, our analysis highlighted within-classroom variability in language experiences in terms of the properties of the CDS addressed to target children and how they were exposed to this input over time. Results are discussed with respect to peer influence on children’s learning, heterogeneity in learning opportunities in classrooms, and the variability of the linguistic environment over time.

## Introduction

There is no doubt that young children learn language through their interactions with others, and that the speech addressed directly to them in the first five years of life is pivotal in this process of development [1]. Children simultaneously draw upon multiple sources of input that contribute to their language acquisition. Their cognitive abilities, such as entrenchment and categorization [2], allow them to detect useful distributional patterns (e.g., word boundaries) and prosodic cues (e.g., stressed syllables) from the language that they hear [3]. Children are also endowed with social-cognitive skills that allow them to learn the meaning of symbolic units based on their communicative function, and to capitalize on non-speech contextual information that accompanies these symbolic units during face-to-face interactions [4].

From this perspective, research on early language acquisition has often focused on the linguistic environments in which children develop, especially the features of language directly addressed to the child, referred to as child-directed speech (CDS). CDS has distinctive features in terms of pitch, intonation, words, and syntactic constructions that stress relevant linguistic information and evokes children’s attention [3]. An ongoing debate concerns the primacy of CDS versus non-direct speech in children’s early language acquisition, with the latter representing speech in the child’s environment that is not directly addressed to the child. Cross-sectional studies in controlled settings find that young children are able to monitor third-party simple conversations and learn new words from the overheard speech [5,6]. These results, however, have not been replicated in naturalistic contexts, where overheard speech is substantially noisier and more cluttered than that in controlled setting. Indeed, studies in naturalistic settings suggest that only linguistic experiences that directly engage children are robust predictors of their future language outcomes. Specifically, based on extensive recordings of infants’ interactions with their families in a typical day at home, Weisleder and Fernald [7] found that adult words directed at children when children were 19 months old, but not overheard speech, were associated with their vocabulary growth at 24 months. Similar results were reported in a study of Mayan children, in which measures of both direct and indirect speech were used to predict children’s vocabulary growth [8]. Such studies suggest that CDS within children’s daily environments is particularly influential to language growth.

Many studies identified features of CDS that were influential to children language development. Considerable evidence points to the importance of quantity of talk, such as the number of words and number of utterances; the more language input children receive, the more opportunities they have to interpret the language, develop precursor skills for vocabulary learning (e.g., segmentation of words and phonological awareness), and practice the processing of linguistic information (e.g., lexical processing efficiency; [9]). The qualitative aspects of talk are also important, such as the diversity of words heard [10]. When children hear more different types of words, they have heightened opportunities to learn new words, but also to hear different phonological patterns and acquire more information about the meaning of new words [11]. Another important feature of CDS is its syntax. Besides promoting the use of different types of sentences in children’s speech [12], different syntactical combinations provide diverse clues about the meaning of words (e.g., the position of the word in a sentence) that children use to learn new words, and facilitates the comprehension of challenging sentences (e.g., passive sentences that require syntactic revision [13]).

The effect of CDS on children’s language development, however, depends to a great extent on the quality of the social context in which language is encountered, especially the synchrony between children and their interactive partners. For instance, Yu, Suanda, and Smith [14] found that the number of labels parents used during play was not associated with their infants’ language growth; however, the number of instances in which the parent and the child were jointly attentive to the item being labeled was significantly associated with vocabulary growth. In line with other studies [15,16], Yu et al. [14] highlighted the importance of synchrony between young children’ attentions and their parents’ attention and verbal behavior as a critical characteristic of social interactions that affect language development, even more than the quantity of speech. Another critical aspect of interactions in language acquisition is back-and-forth conversations. Back-and-forth conversation is a more robust predictor of young children’s language outcomes [17] than quantity of talk exposed to in the home. Thus, understanding children’s linguistic environments requires not only attention to quantity and quality of CDS, but also to the social interactions that children experience, including the exposure to verbal input over time.

Though the home linguistic environment is crucial for children’s language development, early childhood programs, including child care and preschool programs, represent a key context in which many young children are developing their language abilities. According to the National Center for Education Statistics [18], in 2015 38% of 3-year-olds, 67% of 4-year-olds, and 87% of 5-year-olds were enrolled in preprimary programs, and many of these are full-day programs. There is an increasing body of literature focused on the quality of these settings, including the linguistic environment children experience. Such studies largely focus on characteristics of teacher’s language use, such as lexical diversity, sophisticated words, and complex sentences [19,20], as well as strategies to elicit talk from children [21,22]. One recent study, for instance, examined the extent to which preschool teachers used grammatically complex talk and how this evoked complex talk from children [12]. Moreover, previous work showed the relevance of multi-turn conversations in classrooms on child language development. Cabell et al. [21] found that conversations in which teachers use strategies to elicit and extend children’s talk served to accelerate children’s vocabulary knowledge over time.

This body of work may potentially overlook salient characteristics of the preschool classroom as a language-developing context, as it tends to ignore the likelihood that children’s experiences in a classroom are likely highly individualized. Children might differ in the ways by which they elicit language input from the adults with whom they engage, and teachers may also attune their instruction to children at different skill levels [23]. Pelatti et al. [23], for example, examined the duration of language- and literacy- learning opportunities that children were afforded in early childhood classrooms over a 90-minute observational period. The authors found relatively substantial within-classroom variation in children’s exposure to text reading, comprehension of written and oral development, and oral language and discussion. A similar situation may happen with peer interactions, but the literature in this respect is more scarce.

In the present study, we sought to better understand the extent to which preschool-aged children within a single classroom experience diverse language-learning environments in terms of their interactions with teachers and peers and exposure to CDS. Our first goal was to characterize children’s interactions with both teachers and peers, given recent evidence showing that preschoolers’ language growth is influenced by the language skills of their classmates [24,25]. The second goal was to characterize the quantity and quality of the child-directed speech experienced by each child, particularly the overall volume of talk and lexical and syntactic complexity of that talk. In total, three research questions were addressed: (1) To what extent is there variability in the duration and frequency of children’ interactions with their teachers and peers? (2) To what extent is there variability in the linguistic features of children’s interactions including the number of sentences, of different words, and of complex and simple sentences? (3) To what extent is there variability in focal children’ interactions and exposure to child-directed speech over time?

Children’s individual linguistic environment was captured using head-mounted cameras that recorded children’s first-person experiences. The use of this technology allowed us to capture each child’s individual linguistic input from a first-person perspective, as opposed to the perspective of an external observer. Each child wore a head-mounted camera for one hour in the morning on a randomly assigned day during a one-week period. Therefore, children’s video captures transcended a single week in the classroom. Because of the invasive nature of data collection, in that children wore a camera on their head which presumably could capture images and talk by all other adults and children in the classroom, and the intensive coding and transcription of collected data, this study was conducted in one preschool classroom in which all instructional staff (*n* = 3) and the majority (95%) of children’s caregivers provided informed consent.

## Method

### Setting and participants

This study involved human subjects and was approved by the Ohio State University IRB. This study took place in one preschool classroom serving 20 children between the ages of 35 and 60 months in an urban non-profit child-care center. The children in the participating classroom were diverse in SES, race, and ethnicity. The preschool classroom operates on a year-round 10-hour day schedule (7:30 to 5:30) to serve working families, and is staffed by one master teacher and two full-time lead teachers as well as university students to ensure that regulated teacher-child ratio (1:12 for age group of 3 years and 1:14 for age group between 4 and 5 years, [26]) is met across the 10-hour day.

The center contains three preschool classrooms, and one classroom was selected for participation based on a variety of factors, including teacher interest and current ongoing commitments of each classrooms (e.g., participation in other studies). Consent was solicited from the primary caregiver of all 20 children in the classroom, of whom 19 (95%) provided written consent; 15 children had permission to wear the head-mounted camera and 4 were permitted to be in the classroom, be recorded, but not to wear the head-mounted camera. One child did not have permission to participate in the study and was moved to a different classroom during recording times. This procedure was established through collaboration with the teacher and center director. The 15 consented children to wear the head-mounted camera were 47 months on average (Range = 35–58 months) and included 10 boys and five girls. Per caregiver report, 67% were African American, 27% as White, and 7% was not reported. The median of the annual household income of participants’ families was $35,156 (1 un-reported).

### General procedures

Each consented child (focal child) was assigned to wear a wireless head-mounted camera on a randomly assigned day during a four-day period for one hour in the morning and one in the afternoon after nap. Four children simultaneously wore a camera each day, and the master teacher wore a camera at the same time. Note that the master classroom teacher and various research staff wore the cameras in the week prior to their use to help the children become familiar with the technology, to test duration of the battery, and to assess comfort. In this pilot work, we determined that the camera should not be worn for more than one hour because it became heated and caused discomfort. In the process of data-collection, two of the 15 consented children were not represented as one was absent on the days assigned for recording and one child could not wear the head-mounted camera as it could not stably stay in place. Thus, the analyses presented here reflect 13 children’s first-person experiences in their classroom.

Focal children wore a head-mounted camera for one hour in the morning and another hour in the afternoon. However, for the purposes of this study, we elected to analyze only the morning session. In the morning session, children experienced different kinds of activities (whole group activities, transitions, and free choice activities involving multiple centers), therefore representing the typical contextual factors that were associated with the preschool classroom setting [27]. In this study, children wore the head-mounted cameras mainly during circle time, when the entire class convened around a circle to set the stage for the day, and center time, during which children autonomously moved across various activities organized around the classroom.

Footage available for the 13 children averaged 51.11 minutes (range 35.60 to 59 min) during the morning session. Although the range of the recording time across children is relatively large (23.43 min), the recording time of only one child (Child A) is considerably short relative to her peers. We decided to include this child’s data into the analyses since it represents a snapshot of her interaction’s patterns that is comparable with that of her peers during the same activity. Table 1 presents the number of chapter and the recording time per child analyzed in this study. By default, the head-mounted cameras automatically segmented videos into chapters of 12-minute duration. In addition, two children occasionally touched the camera while on their heads, which led to creation of additional chapters. In total, there were 75 chapters for analysis, although some were shorter than 12 minutes. For the present purposes, we included all chapters that were longer than 5 minutes, resulting in analysis of 60 chapters. On average, each child’s video averaged 4.6 chapters (range 3 to 5) and chapters averaged 11.07 minutes in length (range 5.55 to 12 min).

**Table 1:** Number of chapters and recording time per child.

Child	# chapters	Total recording time in min	Averaged recorded time in min (SD)
A	3	35.60	11.87(0.23)
B	5	54.28	10.86(2.07)
C	5	52.70	10.54(2.16)
D	5	52.83	10.57(1.98)
E	4	46.82	11.70(0.27)
F	4	47.48	11.87(0.26)
G	5	59.03	11.81(0.43)
H	5	56.64	11.33(1.18)
I	4	44.28	11.07(1.86)
J	5	53.43	10.69(1.97)
K	5	54.18	10.84(2.47)
L	5	53.55	10.71(2.88)
M	5	53.55	10.71 (1.96)

### Video coding and transcription

Each video chapter was coded and transcribed in its entirety. The coding scheme captured five dimensions of children’s experiences, whereas transcription captured four characteristics of child-directed speech.

Coding was conducted using Datavyu software which provided moment-by-moment documentation of: (a) type of activity, (b) type of play, (c) grouping status, (d) peer interaction, and (e) adult interaction. For the present study, we examined two coded dimensions, namely peer interaction and adult interaction, given our interest in children’s experiences with peer and adults in the classroom; see Table 2 for description of these two dimensions.

**Table 2:** Definitions of the codes used in this study to capture peer and teacher interactions.

Category	Codes	Definition
Peer interaction	Solitary	The student is alone engaging or not in an activity. Peers may be present, but they are engaged in other activities and do not interact with the focal student.
	Reciprocal interaction	The student interacts (e.g., play, work, fight, and discuss) with one or more peers on the same activity *reciprocally* (verbally or physically). To be considered reciprocal, the addressee’s (the focal student or a peer) behavior or speech must occur up to 3 seconds after addresser’s behavior or speech, or her utterances have to have the same topic as the addresser’s preceding utterances.
	Parallel activity	The student works or plays next to, but not with, one or more peers on the same activity, with or without eye contact. The student must be engaged in the same activity as her peer(s); otherwise, the event is coded as *solitary*.
Adult interaction	No interaction	The target child does not interact with an adult.
	Comforting	The teacher comforts the upset or tired child (verbally or physically).
	Discipline	The teacher disciplines the student or solves a problem between the student and peers or between the student and teachers. It includes verbal reprimands.
	Conversation/direction	The student listens to the teacher’s directions, one on one or in a group, or back-and-forth exchanges occur. It includes the teacher giving non-academic instructions and text reading
	Play	The teacher is engaged in playing with the student, with or without peers present, without having control of the activity. If teacher instructs the student (e.g., "put the cars on top and see which one is fastest"), the interaction is coded as *conversation/direction*.

In implementing this coding scheme, the two dimensions were coded in two separate passes through a chapter. Coding was designed to capture when a given event began and ended, thus analyses can examine both event frequency of occurrence as well as duration of events of interest. Peer interaction was captured in the first pass and represented three categories: (a) reciprocal interaction, (b) parallel activity, and (c) solitary. The reciprocal interaction code captured children’s direct interactions with peers, whereas solitary and parallel activity codes represented time when the child was not interacting with peers. Detailed descriptions of these codes appear in Table 2. Adult interaction was captured in the second pass and represented five categories: (a) comfort, (b) discipline, (c) play, (d) conversation/direction, and (e) none. The first four categories captured children’s direct interactions with adults in the classroom, with or without peers present, whereas the fifth category represented time when the child was not interacting with adults (see Table 2).

For transcription, the *Systematic Analysis of Language Transcripts* software (SALT Research Version; [28]) was used to transcribe all chapters verbatim. When transcribing, only the speech directed at the focal child alone or a group of children that included the focal child was transcribed. Thus, the transcribed language from peers and teachers represents “direct talk”. We identified direct talk based on the scheme presented in Fernyhough and Russell [29]: (a) peers and teachers’ behaviors involved the child while they talked (e.g., through physical contact or gaze direction), (b) peers and teachers’ utterances had the same topic as a child’s preceding utterances, was a question directed to the child, or contained the child’s name, or (c) peers and teachers’ utterances occurred up to 3 seconds after the child’s utterance. A peer or teacher utterance meeting any one of these criteria was transcribed.

The transcription process, conducted by the first author and trained, reliable research assistants, followed SALT conventions for segmenting the speech stream into communication units (C-units), as well as for addressing fillers, mazes, and unintelligible and incomplete utterances. Segmentation of running speech into C-units, rather than utterances, utilizes syntactic information for parsing running speech into smaller discrete units: one C-unit consists of one independent clause and all dependent clauses and phrases. When running speech occurred that did not contain any clause structure, these were segmented as if they were a C-unit.

For the purpose of this study, each C-unit and partly intelligible C-units (i.e., C-units that combined intelligible and unintelligible words, such as *go to XXX now* and *In this XXX in Spanish*) produced by the adults and children was coded for complex syntax using the scheme presented in Huttenlocher, Vasilyeva, Cymerman, and Levine ([30]; also used in Justice et al. [12]). Complete unintelligible C-units were not coded. Each C-unit was coded using three mutually exclusive categories: (a) no verb, (b) simple, and (c) complex. *No verb* was applied to units that did not contain verb structures, such as those containing just a noun (e.g., Mommy), a noun phrase (e.g., My hands), or a prepositional phrase (e.g., Up). *Simple* was applied to units that contained a single verb phrase (e.g., I need some red). *Complex* was applied to units that were multi-clausal, containing multiple verb phrases (e.g., I want to sit in that chair).

Within the transcript, every child, teacher, and peer C-unit was also coded for whom it was directed. For C-units attributed to the focal child, the addressee was captured per four mutually exclusive categories applied to each C-unit: (a) child to peer, (b) child to group, (c) child to teacher, or (d) private speech. Transcribed talk affiliated with an adult and captured on a focal child’s transcript was coded as (a) teacher to focal child or (b) teacher to group. Transcribed talk affiliated with a peer and captured on a focal child’s transcript was coded as (a) peer to focal child or (b) peer to group containing the focal child. Table 3 provides definitions for each of these eight codes.

**Table 3:** Definition of codes used to specify the utterances’ addressee in the transcriptions.

Speaker	Addressee code	Definition
Focal child	Peer	Student’s utterances clearly addressed to a peer(s).
	Group of students	Student’s utterances that do not have a specific addressee, but they are followed by a feedback from a member of the group. This code also is used when the child signs songs with her peer(s), and when her interactions with her teacher aimed to send a message to the group where she is (e.g., answering a teacher’s questions to remain all students the rules of an activity).
	Teacher	The student’s utterances clearly addressed to a teacher(s).
	Private speech	Student’s utterances that do not meet any of the following criteria for social speech, adapted from the schema in Fernyhough and Russell [29]: (a) a peer or a teacher sustained eye contact with the focal child during or immediately after child’s utterance, (b) child’s behavior involved a peer or a teacher (e.g., through physical contact or gaze direction) while the child talked, (c) child’s utterances have the same topic as peer’s or teacher’s preceding utterances, or is a question directed to another person, or contained a vocative or another person’s name, and (d) child’s utterances that occur up to 3 seconds after peer or teacher’s social utterance.
Teacher	Focal child	Teacher’s utterances clearly addressed to the focal child.
	Group	Teacher’s utterances addressed to a group where the focal child is. This can be small group, large group or the whole class.
Peer	Focal child	Peer’s utterances clearly addressed to the focal child.
	Group	Peer’s utterances addressed to a group where the focal child is. This can be small group, large group or the whole class.

Multiple steps were taken to ensure the accuracy of the coding and transcription process. First, prior to any coding or transcribing activities, a comprehensive training was completed that included studying relevant materials (e.g., the SALT guide) and multiple practice sessions were completed and compared to gold standards. Second, all transcripts were checked in their entirely for accuracy after the initial transcript was developed. The first author compared the transcript against the video chapter word-by-word to verify that the interaction in which focal children participated were transcribed, and the transcription conventions and syntactic codes were accurately applied. Any ambiguities were annotated and examined with the second author. For the coding activities, coders conducted practice sessions with five chapters, which were compared against master codes created by the first author. In addition, the first author routinely double-coded chapters alongside the coder (for a randomly selected 11% of the overall chapters coded) to monitor and prevent drift. In these double-coding sessions, the two coders discussed any disagreements until consensus was achieved. Finally, an overall reliability estimate was derived from a randomly selected corpus of seven chapters representing approximately 10% of all chapters coded. Chapters that lasted less than 2 minutes (*n* = 4) were excluded from selection; thus, 70 video chapters served as the pool to select the videos for reliability. The process of selection involved first randomly selecting 50% of children in the sample for inclusion and then randomly selecting one chapter from among all of their chapters for double-coding.

Since the occurrence of events (i.e., codes) and its variation during the observed period of time were the main interest in this study, alignment Kappa values and interclass correlations (ICC) were used for reliability purposes. The average alignment kappa values across all chapters were 0.66 for adult interaction (range = 0.42 to 1; *SD* = 0.17) and 0.53 for peer interaction (range = 0.27 to 0.7; *SD* = 0.17). The absolute-agreement 2-way mixed-effects model ICC for the duration of each category was greater than 0.97. Although the alignment Kappa values for the three categories were relatively low, the ICC values for each category suggest excellent reliability. Alignment Kappa values for time-event recording strategy (used in this study) tend to be lower than the classic Cohen’s kappa and single-code alignment kappa, because in the former more commission-omission errors (e.g., one coder recorded an event, the other do not) and more disagreements between observers typically result [31].

## Results

### Children’s interactions with teachers and peers in the classroom

The results are organized around the three questions addressed in this study. The first question concerned the extent to which there is variability in the duration and frequency of children’ interactions with their teachers and peers in which they were exposed to CDS. First, children’s interactions are described using raw values; however, to facilitate the comparison across children, the *frequency* of children’s interactions was normed by time. For example, if a child interacted with others 26 times during a 12-minute video, this was normed to 2 interactions per minute; zero values were retained. Then, we assessed differences among children in these interactions by examining their variability in terms of duration. Finally, we calculated intra-class correlations (ICC) to determine the proportion of variance in the *duration* of interactions between children. Raw values were used in the last two analyses. It is important to bear in mind that though normed data facilitates the comparison across children, this comparison is not completely neat. As explained in the discussion section, the differences in the recording time across focal children might explain to some degree the individual differences reported below.

In general, focal children interacted more frequently and for a longer time with their teachers than their peers. When the total frequency of children’s interactions was observed, on average 60% of these interactions were with teachers (*M* = 39, Range = 25–57 for frequency of teacher interactions, and *M* = 26, Range = 13–46 for frequency of peer interactions). Moreover, the total duration for teacher interaction was, on average, almost three times the total duration for peer interactions (*M* = 75%, Range = 57–90% for duration of teacher interaction, and *M* = 25%, Range = 10–43% for duration of peer interaction). These differences were statistically significant based on the Wilcoxon signed-rank test (*Z* = -2.660, *p* = .008 for frequency of interactions and *Z* = -3.180, *p* = 0.001 for duration of interactions).

**Fig 1:**
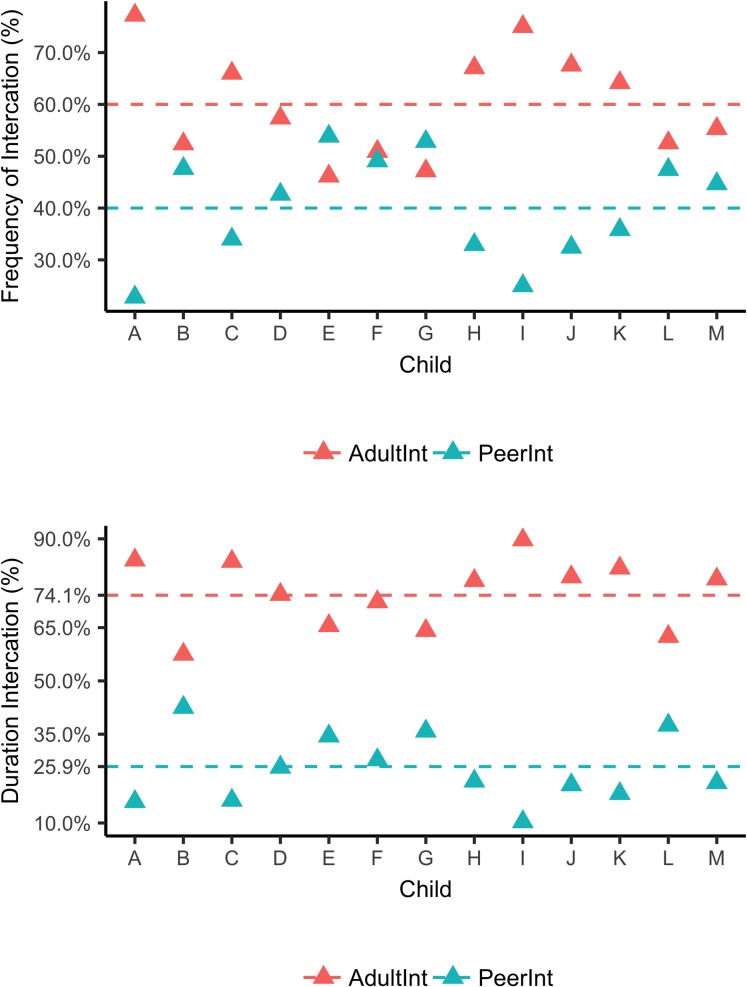
Proportion of the total frequency and duration of teacher and peer interactions per child. The lines represent averaged percentage among all participants.

Focal children, however, differed to some extent from the global tendency, and their first-person experiences revealed interesting individual differences (Table 4 presents descriptive information of focal children’s interactions). Fig 1 presents the proportion of the frequency and duration of teacher (red triangle) and peer interactions (blue triangle) per child. For example, over the total frequency and duration that Child A interacted with both teachers and peers, 23% of the frequency and 17% of the duration of her interactions were with peers. Six of the 13 focal children (Children B, E, F, G, L, and M) interacted with their peers almost as many times as they interacted with their teachers; around 50% of these children’ interactions were with peers. Moreover, relative to the averaged proportion of the duration of peer interactions across all children, four of these children (Children B, E, G, and L) interacted longer with their peers. This information suggests that for some focal children, peers might be an important linguistic source.

**Table 4:** Frequency, median, range, and SD of the duration of focal children’s interactions.

Child	Type of interaction	Frequency (Number) of interactions in 10 min	Duration of interactions (sec)			
			Median	Min	Max	SD
A	Teachers	12.4	9	3	165	28.83
	Peers	3.7	6	3	46	13.02
B	Teachers	6.1	15	3	296	53.82
	Peers	5.5	18	3	138	34.14
C	Teachers	6.3	13	3	297	52.62
	Peers	3.2	11	4	25	6.22
D	Teachers	7.4	17	4	178	35.95
	Peers	5.5	7	3	67	14.76
E	Teachers	6.3	10	3	226	43.71
	Peers	7.3	10	3	43	9.18
F	Teachers	5.9	20	3	217	43.20
	Peers	5.7	8	2	55	12.81
G	Teachers	4.2	14	4	367	77.37
	Peers	4.7	17	5	85	17.51
H	Teachers	10.1	10	3	238	32.95
	Peers	4.9	8	3	60	10.78
I	Teachers	10.8	20	2	198	40.71
	Peers	3.6	10	4	39	8.56
J	Teachers	9.4	21	2	234	38.21
	Peers	4.5	10	2	117	24.57
K	Teachers	7.9	22	3	220	38.74
	Peers	4.4	12	3	35	9.00
L	Teachers	9.5	10	3	228	35.22
	Peers	8.6	10	3	63	13.16
M	Teachers	4.9	25	4	232	44.91
	Peers	3.9	12	3	32	8.05
Average	Teachers	7.23	16	3	238	43.56
	Peers	5.04	11	3	61	13.98

*Note*: Teacher = Teacher interaction; Peer = Peer interaction. The frequency of children’s interactions was normed by 10 minutes to facilitate the interpretation of the data.

**Fig 2:**
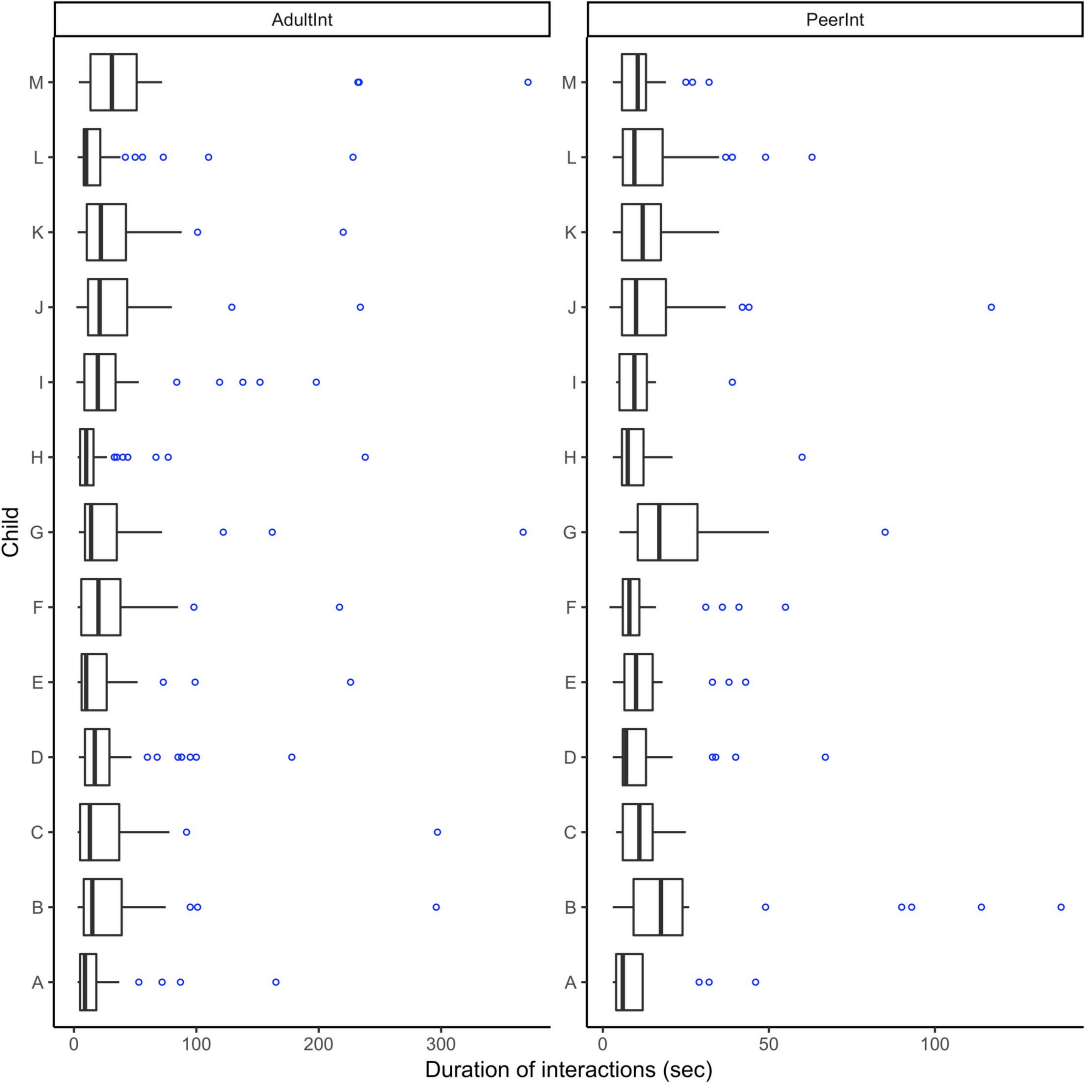
Distribution of the durations (in seconds) of teacher (left) and peer interactions (right).

When the duration of children’s interactions with teachers and peers was examined, a noteworthy characteristic was the high variability of the interactions. Fig 2 presents the distribution of the duration in seconds of discrete interactions per each child. The central 50% for teacher interactions was between 7.3 seconds (Range = 5–11.5 sec., *SD* = 2.28 sec) and 30.2 seconds (Range = 16–43.5 sec., *SD* = 9.33), on average (IQR range = 11–32 sec.). The central 50% of peer interactions was between 6.35 (Range = 4–10.5 sec., *SD* = 1.70 sec) and 16.26 seconds (11–28.5 sec., *SD* = 5.14) on average (IQR range = 5–18 sec.). As Fig 2 shows, the majority of children had interactions for which the duration was substantially larger relative to the tendency of the duration of their interactions (i.e., above the result of third quartile plus 1.5 times IQR). This is particularly evident in focal children’s interactions with their teachers.

This information suggests that children experienced very different types of interactions, in terms of duration, during the recorded time. To answer the question of how much the total variance in the duration of children’s interactions is due to differences among children, we calculated ICC using data representing discrete interactions. We reported ICC per every chapter (5 chapters in total) for two main reasons: children were observed in different activities (circle time and center time) and the number of chapters in both activities was different across children; by calculating ICC per chapter instead of overall, we control for differences in video footage length during the same activity. These results are presented in Table 5. Since children differed to some extent in the recorded time, not all children had the same number of chapters. Therefore, the number of children per chapter is different.

**Table 5:** ICC Values for the duration of focal children’s interactions, per chapter.

Chapter (number of children)	Teacher interaction	Peer interaction
1 (N = 12)	0.00	0.19
2 (N = 13)	0.13	0.00
3 (N = 13)	0.06	0.13
4 (N = 13)	0.00	0.15
5 (N = 9)	0.00	0.05

The results of ICC calculations suggest individual differences in the duration of children’s interactions. In the chapters that corresponds to circle time activity, approximately 19% of the variance observed in the length of peer interactions is explained by differences among children (ICC = 0.19). The variance in the duration of teacher interaction in this activity was comparable among children (i.e., ICC values were close to zero). Individual differences were notable in some chapters for center time. For example, in one chapter of this activity, approximately 13% and 15% of the variance in the duration of teacher (ICC = 0.13) and peer (ICC = 0.15) interactions, respectively, was due to individual differences among focal children. The ICC values very close to zero in some chapters of center time suggest that children also had different interactions, in terms of durations, over time.

### Children’s linguistic experiences in the classroom

While the previous results described the frequency and duration of the interactions during which focal children were exposed to language input, we now present the linguistic properties of these interactions from children’s first-person perspective. The second research question sought to determine the extent of the variability in the linguistic features of children’s directed-speech, including the number of sentences, different words, and complex and simple sentences. To answer this question, first we examined the characteristics of the CDS addressed to focal children and compared these characteristics across children. To do so, we normed our counts by recording minutes. For example, if a child heard 139 different words in 12 video-minutes, this was normed to 12 different words per minute; zero values were retained. Additionally, to facilitate the comparison across children, we used the Hodges Lehmann’s median across chapters *per child*. This provides an estimator of the center of non-symmetric distributions, like the distribution of the linguistic measures of this study. Finally, we calculated ICC to determine the proportion of variance in the *counts of the linguistic properties of the CDS* between children. We used the time-normalized data in this calculation. As mentioned in the previous section, though normed data allow us to compare the information across different children, this comparison is not completely free from error due to the differences in the length of children’s videos.

On average, the majority of linguistic input directly addressing the individual children (or a group where they were) came from teachers: 81% of sentences (Range = 66–95%), 89% of different words (Range = 80–98%), 92% of complex sentences (Range = 73–100%), and 82% of simple sentences (Range = 72–94%) originated from teachers, on average. Table 6 presents the time-normed count measures that each child heard from teachers and peers.

**Table 6:** Median and SD of the measures of the CDS addressed to focal children.

Child	Number of utterances		Number of different words		Number of simple sentences		Number of complex sentences	
	From teachers	From peers	From teachers	From peers	From teachers	From peers	From teachers	From peers
	Median (SD)	Median (SD)	Median(SD)	Median (SD)	Median(SD)	Median(SD)	Median(SD)	Median(SD)
A	7.9(2.8)	0.4(0.3)	46.4(13)	1.1(0.7)	4.1(1.6)	0.2(0.1)	2.8(0.7)	0
B	6(4.8)	1.9(1.6)	30.6(29.2)	5(3.8)	3.2(2.8)	1(0.7)	1.7(1.2)	0.1(0.1)
C	5.1(5.4)	1.1(0.6)	23.6(28.7)	3.3(1.9)	3.4(2.8)	0.6(0.3)	1.3(1.5)	0.1(0)
D	8.7(5.6)	2.6(1.3)	51.4(28.8)	7.6(4.1)	4.3(3.1)	1.6(0.9)	3.2(1.4)	0.2(0.2)
E	2.5(5.6)	1.7(3)	15.2(35.1)	5.1(8.6)	1.6(3.3)	0.9(1.2)	0.8(1.4)	0.5(0.6)
F	6.9(5.1)	1.9(0.5)	36.9(27.1)	6.8(1.7)	3.9(3.1)	1.2(0.4)	2(1.5)	0.3(0.1)
G	6.3(5.4)	1.5(1.1)	34.3(34.1)	3.8(5)	3.6(3.3)	1(0.8)	1.5(1.6)	0.4(0.2)
H	4.9(4.5)	1.4(0.6)	25.5(32.4)	3.7(1.5)	2.8(2.8)	0.9 (0.5)	1.5(1.6)	0.2(0.2)
I	11.3(6.1)	0.8(0.1)	68.6 (39.7)	2(0.4)	6.7(3.5)	0.5(0.1)	3.5(2.2)	0.2(0.1)
J	10.4(4.3)	2.1(1.6)	58.9(22.4)	6.2(5.1)	6(2.7)	1.4(0.9)	3.1(0.7)	0.2(0.1)
K	10.1 (5.1)	0.8(0.6)	53.3(26.6)	2.2(2.3)	5.5(2.9)	0.5(0.2)	3(1.2)	0.2(0.1)
L	6.3(5.5)	1.9(0.8)	35.5(42.6)	6(2.9)	3.7(3.7)	1.1(0.5)	1.8(1.4)	0.2(0.2)
M	3.7 (6.2)	1.3(0.8)	21.2(35.9)	4(2.7)	2.1(3.3)	0.8(0.5)	1.3(1.7)	0.3(0.2)
Average	6.9(5.1)	1.5(1)	38.6(30.4)	4.4(3.1)	3.9(3)	0.9(0.6)	2.1(1.4)	0.3(0.2)

*Note*: the median values correspond to Hodges Lehmann’s median across chapters. The counts measures were normed by 10 min to facilitate the interpretation of the data.

**Fig 3:**
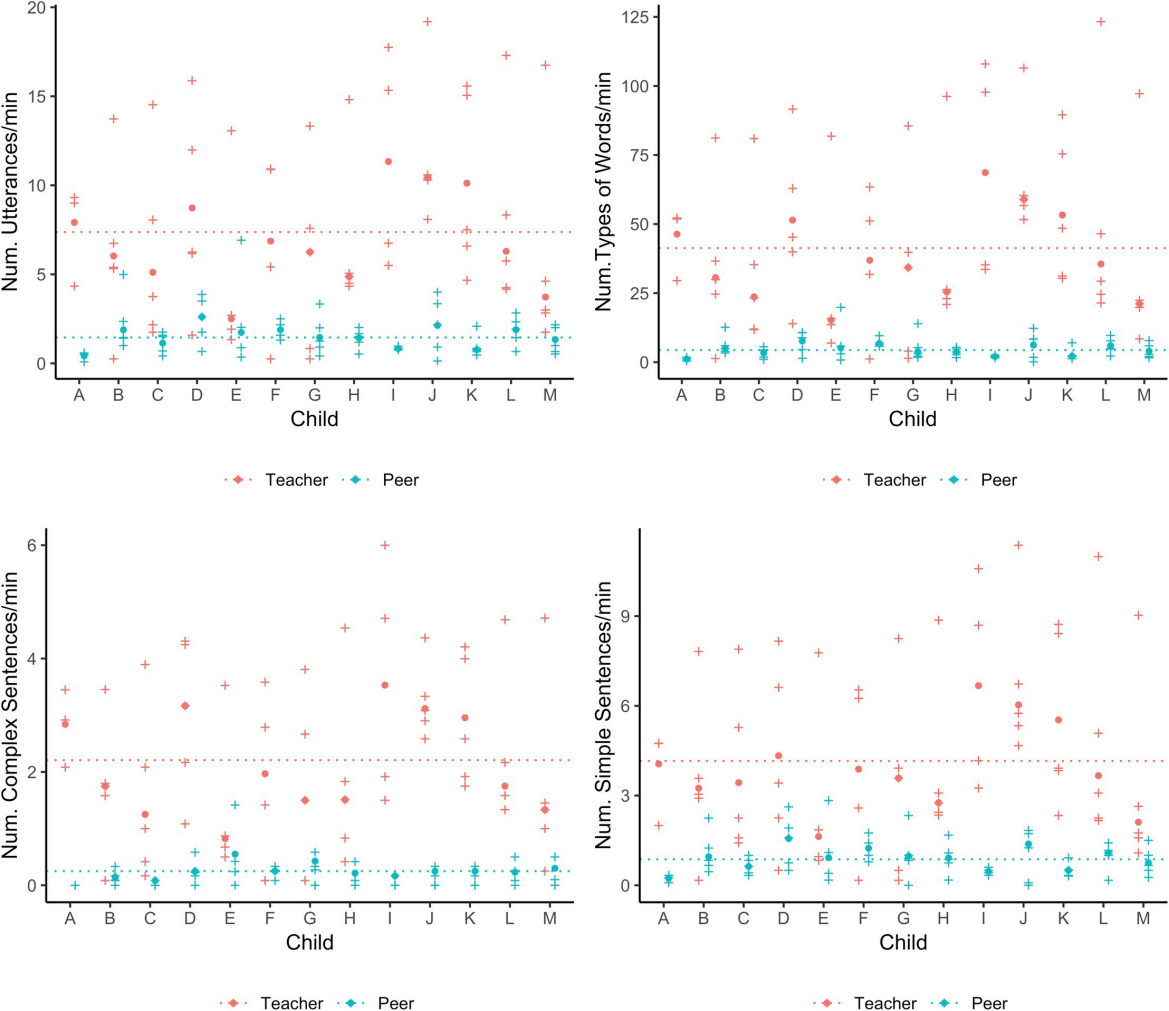
Number of utterances, of word types, complex sentences, and simple sentences directed to the participants from teachers and peers. The crosses represent the values in each video chapter and the dots the Hodges Lehmann’s median. The lines represent averaged percentage among all children.

More detailed analyses of the linguistic environment, however, revealed high variability both within children’s own linguistic experiences across the recorded time, as well as differences among children. Fig 3 presents the time-normalized number of sentences, types of words, complex sentences, and simple sentences that each child heard from peers (blue cross) and teachers (red cross) in each chapter. The big dot corresponds to the Hodges Lehmann’s median across the chapters for each child. Each cross represents the number of linguistic properties of interest (e.g., counts of utterances) per minute in each chapter.

As expected, we observed substantial variability in children’s linguistic input over time (across chapters), even during the same type of activity. For instance, according to the normalized data, for every utterance per minute that Child B heard from peers in one chapter of center time, the same child heard 5 utterances per minute from peers in a different chapter of the same activity. This variability was more notable in the linguistic properties of the speech provided by teachers to individual children. During center time, for every utterance per minute that teachers addressed to Child B (or group where she was) in one chapter, the teachers addressed 27 utterances per minute in a different chapter. Thus, according to these normalized values, in the first 10 minutes of center time this child could hear around 10 utterances from teachers, and by the end of the activity the same child could hear 270 from them.

Based on the Hodges Lehmann’s median, Fig 3 also shows that some children tended to have more verbal interactions with their teachers and peers than others. For example, for every type of word and complex sentence that Child E heard per minute from teachers, Child I heard 5 and 4 respectively. Thus, in ten minutes Child E could hear around 10 different words and 10 complex sentences from teachers, while Child J could hear around 50 different words and 40 complex sentences from teachers. Likewise, for every type of word that Child A heard per minute from peers, Child F heard 6, and for every complex sentence that Child C heard per minute from peers, Child G heard 5.

We next sought to determine how much of the total variance in the counts of the linguistic properties of speech addressed to focal children is due to differences among children as opposed to differences over time. To do so, we calculated an ICC using time-normalized counts of the linguistic properties of speech heard by children. Note that unlike the previous analyses with interaction duration which partitioned variance at the level of each discrete interaction, the linguistic information used to estimate variance differences for the linguistic properties of speech was collected per chapter. One chapter was contained circle time activity in which all children are experiencing the same linguistic input from teachers and peers. Thus, we calculate the ICC including all chapters and the ICC excluding the chapter that corresponded to circle time.

When all chapters were included in the analyses, the results of the ICC showed that the majority of the variance of the properties of the focal children’s CDS was attributable to differences across time. In the speech heard from teachers, only 3% of the variance in the number of complex sentences was attributable to differences between children (ICC = 0.028). For the rest of the linguistic properties (number of utterances, types of words, and simple sentences from teachers) the variance of the data across all children was comparable (i.e., ICC values were very close to zero). The variance attributable to children was similarly small in the properties of the speech that focal children heard from peers. Only 2% of the variance of the number utterances (ICC = 0.025), 3% of the variance of number of types of words (ICC = 0.028), and 2% of the variance of the number of simple sentences (ICC = 0.019) were attributable to differences between children. Nonetheless, differences among focal children explained a higher proportion of the variance of the number of complex sentences heard from peers (7%, ICC = 0.069).

We see important differences when the chapter that corresponded to circle time was excluded from the ICC calculations. This new result suggests individual differences in the child-directed speech that children heard, especially from teachers. In the speech that focal children heard from teachers, the ICC was approximately 22% for the number of utterances (ICC = 0.217), 33% for number of types of words (ICC = 0.333), 31% for the number of complex sentence (ICC = 0.306), and 18% for the number of simple sentences (ICC = 0.178). Unexpectedly, the child-directed speech provided by peers becomes more comparable across all children (i.e., ICC values were very close to zero).

### Patterns of focal children’ exposure to directed speech over time

The third question concerned the variability of focal children’ interactions during the recorded time, and therefore the exposure of directed speech over time. For this purpose, we compared the total duration that focal children interacted with others versus the duration of no interaction (i.e., when children did not interact with others). Moreover, we describe the regularity in which focal children interacted with teachers and peers over time.

Though focal children significantly interacted more with their teachers and, therefore, heard more speech from them than from peers, they did not interact with their teachers all the time. The averaged proportion of the total duration of teacher interaction over the length of the recording time was 36% (Range = 28–55%), and the averaged proportion of peer interaction over the length of the recording time was 12% (Range = 6–25%). These differences between time of interaction and time of *no* interaction were statistically significant according to Wilcoxon signed-rank test (Z = -2.970, *p* = .003 for the difference between duration of teacher interaction vs duration of no teacher interaction, and Z = -3.180, *p* = .001 for the difference between the duration of peer interaction vs the duration of no peer interaction). Together, these results suggest that, from children’s first-person perspective, long instances of interactions and, therefore, long exposure to CDS was more the exception than the rule. Focal children had bouts of interactions with others and, thus were exposed to bouts of CDS over time.

**Fig 4:**
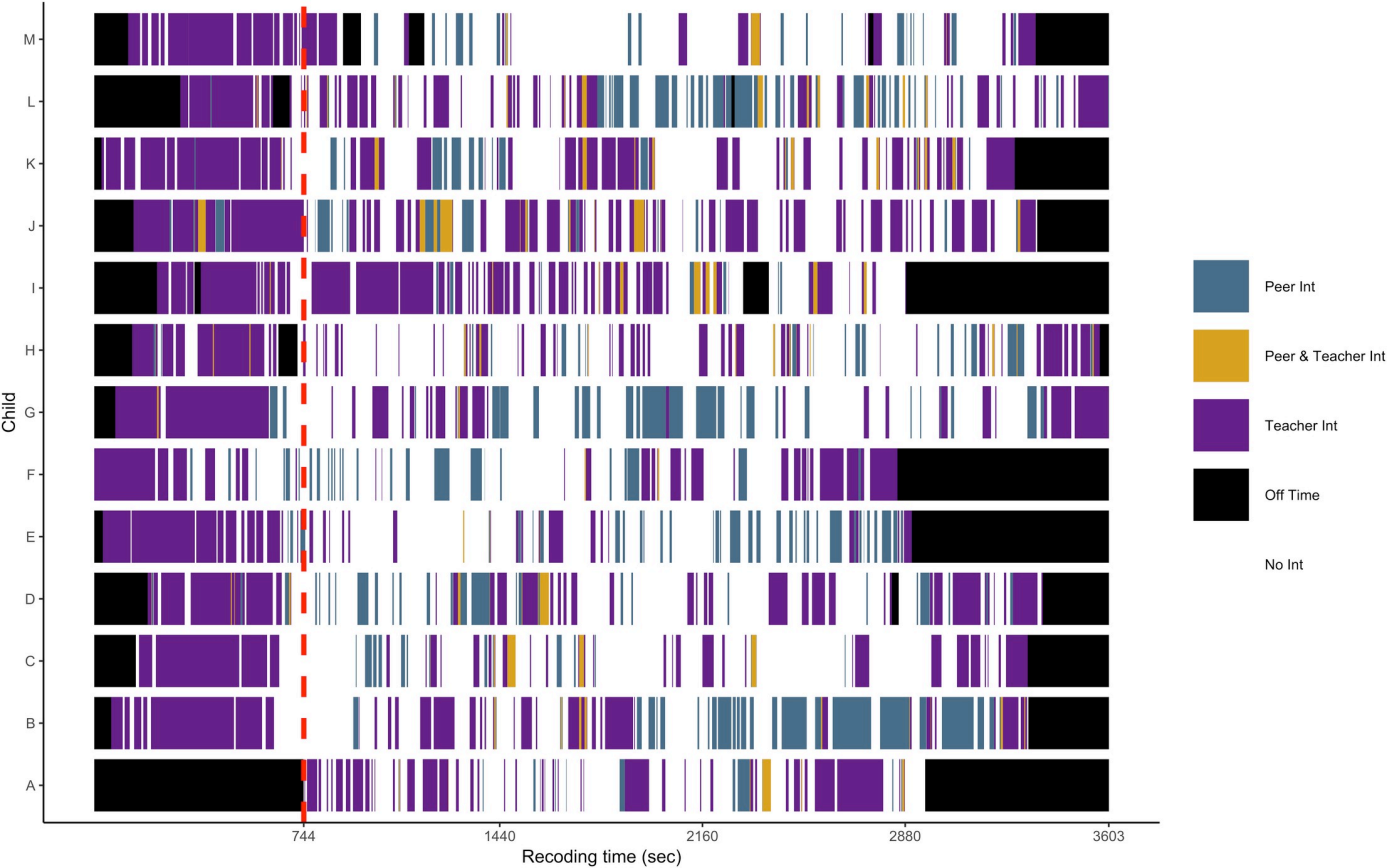
Sequence of focal children’s interactions over the recording time. The dotted line demarcates when the center time starts. Off Time corresponds to the instances when the head-mounted camera was off.

To what extent children differed in their regularity of interactions with others, and therefore, in their opportunities to linguistically interact with others? Fig 4 presents a timeline of focal children’s interactions with their teachers, peers, and with both simultaneously (e.g., when a teacher helped a focal child to make a plan with her peer to use a toy) over the total recording time. The red dotted line divides the timeline into the types of activities; the first segment corresponds to circle time and the last to center time. The heterogeneity in children’s verbal interactions was more salient during center time. Some focal children, such as the cases I and J, had more constant and longer interactions with their teachers than other focal children, like cases E and M. Similarly, some focal children, such as the cases B and L had more frequent and longer interaction with peers than other children, such as the cases C and H. Interestingly, the majority of the instances of peer interaction took place without the mediation of the teachers. Thus, children differed not only in terms of the frequency and durations of interactions with others, and in the features of the language addressed to them, but also in how these experiences occurred over time.

## Discussion

This study offers a new perspective of the linguistic environment of preschool classrooms by documenting preschoolers’ first-person perspectives of their interactions with others and exposure to child-directed speech. The substantial increase in the number of young children who attend preschool settings has heightened researchers’ attention toward the quality of these settings, which has been frequently described and assessed at the system level [19,32]. Nonetheless, a nascent line of research has found that children undergo different learning experiences within the classroom [23]. This study contributes to this line of research: it explored the individual differences in the CDS addressed to preschoolers within a classroom. This study used an innovative emerging technology in the classroom setting that captured the perspective of the learner: head-mounted cameras. Earlier work has shown that the first-person view creates functional patterns in terms of visual properties and access of social clues that are not captured by a third-person perspective [33]. Thus, Yurovsky et al. [33] suggested that the mechanism by which the environment affects children’s development is through the linguistic experiences with which children have direct contact, making our study a particularly salient examination of the linguistic experiences that might influence children’s language development in the preschool setting. Below, we discuss four major findings and their implications for further research.

First, when the interactions in which children experienced CDS are observed from children’s first-person perspectives, one noticeable result was the variability in their number and duration; the average values in this respect did not represent every child’s experiences. Relative to the averaged number and duration of teacher and peer interactions, some preschoolers interacted less frequently and for less time with their teachers, but more frequently and for longer with their peers. This suggests that preschoolers from the same classroom might undergo different linguistic experiences; in our study, for some children the main source of CDS during the observed period of time was their teachers while for others their peers served as an additional source of this verbal input. It was interesting, for example, that two children (E and M) tended to hear a similar number of sentences per minute from their peers and teachers. Our finding converges with those of Perry et al. [34], who found that in a classroom some children tended to be more exposed to peers’ and teachers’ talk than others. These authors suggested that children’s language skills may contribute to the explanation of this variability. The current study extends these findings by describing the CDS’s features that peers and teachers directly addressed to each focal child or to the group where they were.

A second finding that was striking was how brief peer interactions were, and how simple peer’s linguistic input was relative to teachers’ linguistic input. A similar trend was documented by Test and Cornelius-White [35], who highlighted the role of preschoolers’ social interaction timing in children’s engagement in activities. Using the pooled data of all participants, the authors found that the total time of different types of child-peer talk (e.g., talk on topic or talk in a new activity) was up to 2.9% of the total observed time (192.7 min; each participant was observed 16 min on average). In spite of its brevity, and unlike teacher interactions, the authors found that peer interactions facilitated children’s continuing engagement in an activity over relatively long period. In this line, and keeping in mind that the current study coded and transcribed *reciprocal* peer interactions, we could hypothesize that the linguistic input during brief but engaging peer interactions, as the linguistic patterns found in the current study, might shed lights on the mechanism whereby peer affect children’s language development. Further research is needed to investigate how young children benefit from peers’ talk and what contextual factors facilitates conversational turns among children.

When someone passes by a preschool classroom, it is almost impossible to ignore the multiple voices that come from it. But how much of this language directly engages each child in the classroom? The third main finding of this study suggested that children’s linguistic experiences in the classroom might be individualized. Previous literature thoroughly described teachers’ language use during structured vs unstructured activities [22], different contents of instruction [19], and different book genres [36]. The current study, however, suggests that when these settings are examined from children’s first-person perspectives, every child might undergo very different linguistic inputs and, therefore, different learning opportunities. This was particularly evident during center time, a type of activity that represents much of the preschoolers’ time in a school day, relative to other activities [27]. In this study, the CDS addressed to every child in the classroom when they were engaged in the same activity in the same period of time was qualitatively and quantitatively distinct, and average values across all participants did not accurately represent each child’s linguistic experiences. This was particularly notorious in the verbal input originated from teachers.

Previous studies have documented the heterogeneity of children’s learning opportunities within a classroom [23] and, more interesting, how these individual experiences differ from the global quality of the classroom [32]. Jeon et al. [32] compared the quality of young children’s individual experiences with the global quality of the classroom they attended, by using a measurement that assessed processes that directly affect children’s development, such as teacher-child interaction. The authors found that 62% of the 47 students who attended a “good-quality” classroom were rated as having mediocre-quality individual experiences, and that 33% of the 51 children who attended a “mediocre-quality” classroom was rated as having poor-quality individual experiences. Thus, and as the current study suggests, although teachers’ language use in a setting has particular features, the CDS that children actually hear in the same setting might have different features.

Together, these findings highlight the need for further investigation of the linguistic variability within a classroom and its effects on children’s language learning. Teachers may adapt their teaching strategies to their students’ learning needs and, therefore, children may undergo different learning opportunities in the classroom. It is also possible that children evoke different learning opportunities based on individual characteristics [32,23]. Perry et al. [34], for instance, found that young children who vocalized more tended to be more exposed to verbal input from peers and engaged in conversational turns with their teachers. By examining the individual experiences within a classroom, we can better understand how classroom-based interventions and teacher-language-enhancing interventions reach individual children, and why some of them do not have the expected impact on children’s development [37,38].

Finally, our findings revealed that the classroom’s linguistic environment might be highly fluctuating during the day. In this study, preschoolers were not exposed to CDS during all of the recorded time, but to small bouts of this verbal input over time; the median of teacher and peer interactions with the focal child lasted on average 14.7 seconds (Range = 9–24.5 secs) and 10.54 seconds (Range = 6–17.5 secs), respectively. Importantly, it was found that the characteristics of this verbal input varied over time, even during the same type of activity. Earlier work showed that teachers’ language use is subject to contextual factors, children’s skills, and educational and professional background. The majority of them, however, have not documented timing features of children’s verbal interactions. This information might be a relevant factor in providing responsive and stimulating learning opportunities in a classroom [35]. Studies on parent-child dyads have emphasized the time synchronization of their attention and behaviors in children’s vocabulary learning (14–16). For instance, Yu and colleagues [14] found that the critical characteristic of parents’ responsivity that boosts young children’s word learning is the temporal window between children’s sustained attention to the named object and hearing the object’s label: in successful naming events, parents provide the label as soon as child attend to the named object. In early educational settings, one defining feature of high-quality instruction is extended teacher-child conversations, which comprises multiple turns [39]. Extended conversations sustain children’s attention on a topic and facilitate opportunities for teachers to use new and semantically related words [21]. Hence, the language addressed to young children per se may not be as critical for their language acquisition as the verbal input that they hear in long and responsive conversations with others. Cabell et al. [21] found that the more concentrated conversations the teacher had with young children (i.e., use of multiple strategies that expanded and elicited children’s talk in few conversations) the greater children’s vocabulary gains were over time. Though the relation between multiple turns in discrete conversations and their duration is beyond the scope of this study, long conversations are scarce in early educational settings [21] and, as the current research suggests, this may be especially true for some preschool children from the same classroom. In this study teacher-child interactions were both very short and quite long. Future studies should investigate how this quality of the preschool classroom’s environment influences child language development, and how it facilitates or hinders peer interactions.

To sum, this study examined the individual variability in the kind of linguistic input addressed to children that boosts their language learning using an innovative instrument in the classroom setting: head-mounted cameras. This study’s findings provide initial evidence that the linguistic environment of a preschool classroom is highly dynamic from the perspective of the learner. Nonetheless, it is important to interpret them in the context of some limitations. First, the classroom was captured for each child during only one day. One-day observation is frequent in educational research due to the considerable expense to collect and code the observational data [23]. Although children’s interactions were observed in a typical school day for a relatively long period of time in different activities, the patterns documented here might not be systematic from day to day; in other words, it is possible that the reported results are not representative of children’s experiences across different days or different times during the school day. This study does reveal the variability of the linguistic environment of the classroom when it is observed from their first-person perspective. Future studies using a similar method should use better sampling methods of language input and, as one of the reviewers suggested, ask caregivers about the representativeness of children’s behaviors during the observed time. Second, focal children differed in the total recording time. Although we normed the data by time to make comparisons across children, which is a common research practice to deal with videos with different lengths, it is possible that the differences in the recoding time across children influenced to some extent the individual differences documented in this study. In comparison to shorter videos, longer videos may capture more interactions, but also more different types of interactions when there is more than one actor to whom interact with (teacher and peers). This is likely since an inverse relation between child-teacher and child-peer interactions tends to occur; if a child interacts with the teacher, she is not available to interact with her peers simultaneously. We did not truncate the duration of all videos to the duration of the shortest one because this significantly reduces the data available for analysis. This change involves excluding the information of 18% to 40% of the recorded time per child. Note, however, that with the exception of one case (Child A), the differences between the total recorded time across children were relatively small.

Third, the small sample size, the small variability in participants’ cultural and socioeconomic background, and the characteristics of the child-care centers threat the external validity of the study. The patterns of interactions of the largely African-American group of children with a relatively high teacher-child ratio may not be typical of other classroom settings. Furthermore, the activities and teaching strategies in classroom of university-affiliated centers might not represent the typical activities in other types of child-care centers. Examining the linguistic environment that surrounds children helps us to understand what they could learn. Through examining their first-person perspectives–tied to their body and their momentary disposition in space [40]–we can better understand how and from which experiences they learn from that, as this study suggested, may vary from child to child.
